# Supplementary material for: Comparison of Different Machine Learning Classifiers for Glaucoma Diagnosis Based on Spectralis OCT
Source: Diagnostics (Basel). 2021 Sep 19;11(9):1718. doi: 10.3390/diagnostics11091718 (PMC8471622; doi:10.3390/diagnostics11091718)
Supplement: Supplementary file 1 [file diagnostics-11-01718-s001.zip › diagnostics-1362707-supplementary.pdf]

Table S1. All variables used in the machine learning classifiers

**cRNFL** (n=7)	**BMO-MRW** (n=7)	**ETDRS** (n=36)		**PPAA** (n=64)			
cRNFL_T	MRW_T	mFT_T1	GCL_T1	rFT_11	rFT _31	rFT _51	rFT _71
cRNFL_TI	MRW_TI	mFT _T2	GCL _T2	rFT _12	rFT _32	rFT _52	rFT _72
cRNFL_NI	MRW_NI	mFT _I1	GCL _I1	rFT _13	rFT _33	rFT _53	rFT _73
cRNFL_N	MRW_N	mFT _I2	GCL _I2	rFT _14	rFT _34	rFT _54	rFT _74
cRNFL_NS	MRW_NS	mFT _N1	GCL _N1	rFT _15	rFT _35	rFT _55	rFT _75
cRNFL_TS	MRW_TS	mFT _N2	GCL _N2	rFT _16	rFT _36	rFT _56	rFT _76
cRNFL_G	MRW_G	mFT _S1	GCL _S1	rFT _17	rFT _37	rFT _57	rFT _77
		mFT _S2	GCL _S2	rFT _18	rFT _38	rFT _58	rFT _78
		mFT _C	GCL _C	rFT _21	rFT _41	rFT _61	rFT _81
		mRNFL_T1	IPL_T1	rFT _22	rFT _42	rFT _62	rFT _82
		mRNFL _T2	IPL _T2	rFT _23	rFT _43	rFT _63	rFT _83
		mRNFL _I1	IPL _I1	rFT _24	rFT _44	rFT _64	rFT _84
		mRNFL _I2	IPL _I2	rFT _25	rFT _45	rFT _65	rFT _85
		mRNFL _N1	IPL _N1	rFT _26	rFT _46	rFT _66	rFT _86
		mRNFL _N2	IPL _N2	rFT _27	rFT _47	rFT _67	rFT _87
		mRNFL _S1	IPL _S1	rFT _28	rFT _48	rFT _68	rFT _88
		mRNFL _S2	IPL _S2				
		mRNFL _C	IPL _C				

cRNFL: circumpapillary retinal nerve fiber layer; BMO-MRW: Bruch's membrane opening opening-minimal rim width; ETDRS: early treatment diabetes retinopathy study; PPAA: posterior pole asymmetry analysis; mFT: macular full thickness; mRNFL: macular retinal nerve fiber layer; GCL: ganglion cell layer; IPL: inner plexiform layer; rFT: retinal full thickness; T: temporal; TI: inferotemporal; NI: inferonasal; N: nasal; NS: superonasal; TS: superotemporal; G: global; C: central; S: superior; I: inferior

Table S2. Variable importance ranking using the five machine learning classifiers for all glaucoma eyes (only the first 15 important variables shown)

Rank	CIT	LMT	C5.0	RF	XGBoost
1	cRNFL_G	cRNFL_G	GCL_T2	GCL_T2	GCL_T2
2	GCL_T1	GCL_T1	MRW_TI	cRNFL_G	cRNFL_G
3	GCL_T2	GCL_T2	cRNFL_TS	GCL_T1	GCL_T1
4	MRW_G	MRW_G	cRNFL_NS	MRW_G	MRW_G
5	MRW_TI	MRW_TI	GCL_I1	GCL_I1	cRNFL_TS
6	GCL_I1	GCL_I1	mRNFL_S2	cRNFL_TS	GCL_I1
7	cRNFL_TS	cRNFL_TS	cRNFL_TI	MRW_TI	MRW_TI
8	MRW_TS	MRW_TS	GCL_T1	MRW_NI	MRW_NI
9	cRNFL_TI	cRNFL_TI	MRW_N	IPL_T2	MRW_N
10	MRW_NI	MRW_NI	MRW_G	cRNFL_TI	MRW_T
11	IPL_I1	IPL_I1	rFT_33	MRW_TS	MRW_TS
12	GCL_S2	GCL_S2	mRNFL_I1	rFT_28	rFT_28
13	rFT_28	rFT_28	cRNFL_G	GCL_I2	rFT_88
14	IPL_T1	IPL_T1	rFT_88	mRNFL_I2	IPL_T2
15	GCL_S1	GCL_S1	IPL_T1	MRW_T	rFT_33

CIT: conditional inference trees; LMT: logistic model tree; RF: random forest; XGBoost: extreme gradient boosting; AUC: area under the receiver operating characteristic (roc) curve

Table S3. Variable importance ranking using the five machine learning classifiers for early glaucoma eyes (only the first 15 important variables shown)

Rank	CIT	LMT	C5.0	RF	XGBoost
1	GCL_T2	GCL_T2	GCL_T2	GCL_T2	GCL_T2
2	cRNFL_G	cRNFL_G	cRNFL_TS	cRNFL_G	GCL_T1
3	GCL_T1	GCL_T1	MRW_TI	GCL_T1	cRNFL_G
4	MRW_G	MRW_G	mRNFL_S2	MRW_G	MRW_G
5	MRW_TI	MRW_TI	cRNFL_NS	cRNFL_TS	cRNFL_TS
6	cRNFL_TS	cRNFL_TS	GCL_T1	GCL_I1	GCL_I1
7	GCL_I1	GCL_I1	MRW_G	MRW_TS	MRW_TI
8	MRW_TS	MRW_TS	MRW_N	MRW_TI	IPL_T1
9	IPL_T1	IPL_T1	mRNFL_S1	IPL_T2	rFT_88
10	GCL_S2	GCL_S2	rFT_88	MRW_NI	MRW_TS
11	IPL_T2	IPL_T2	GCL_I1	IPL_T1	rFT_23
12	IPL_I1	IPL_I1	mRNFL_I1	rFT_28	MRW_N
13	MRW_NI	MRW_NI	GCL_S2	IPL_I1	IPL_T2
14	GCL_S1	GCL_S1	IPL_T1	MRW_N	MRW_NI
15	MRW_N	MRW_N	IPL_N1	MRW_NS	rFT_28

CIT: conditional inference trees; LMT: logistic model tree; RF: random forest; XGBoost: extreme gradient boosting; AUC: area under the receiver operating characteristic (roc) curve

Table S4. Variable importance ranking using the five machine learning classifiers for moderate glaucoma eyes (only the first 15 important variables shown)

Rank	CIT	LMT	C5.0	RF	XGBoost
1	GCL_T1	cRNFL_G	GCL_T2	GCL_T2	GCL_T2
2	cRNFL_G	GCL_T1	MRW_N	cRNFL_G	cRNFL_G
3	GCL_T2	GCL_T2	cRNFL_G	MRW_G	MRW_G
4	GCL_I1	GCL_I1	GCL_T1	GCL_T1	GCL_T1
5	cRNFL_TI	cRNFL_TS	MRW_G	IPL_T2	MRW_N
6	cRNFL_TS	cRNFL_TI	cRNFL_TS	cRNFL_TS	cRNFL_TS
7	mRNFL_I2	MRW_G	cRNFL_NS	MRW_NI	MRW_NI
8	IPL_T2	IPL_T2	mRNFL_I2	mRNFL_TI	GCL_I1
9	GCL_N1	NFL_I2	MRW_NS	GCL_I1	IPL_T2
10	MRW_G	MRW_NI	cRNFL_TI	MRW_TI	cRNFL_T
11	IPL_I1	MRW_TI	GCL_I1	MRW_N	cRNFL_TI
12	MRW_TI	IPL_I1	mRNFL_I1	mRNFL_I2	rFT_86
13	MRW_NI	GCL_N1	MRW_TI	MRW_NS	mRNFL_I2
14	IPL_T1	GCL_S1	cRNFL_NI	MRW_T	MRW_T
15	GCL_S1	GCL_S2	rFT_54	rFT_28	rFT_33

CIT: conditional inference trees; LMT: logistic model tree; RF: random forest; XGBoost: extreme gradient boosting; AUC: area under the receiver operating characteristic (roc) curve

Table S5. Variable importance ranking using the five machine learning classifiers for severe glaucoma eyes (only 15 most important variables shown)

Rank	CIT	LMT	C5.0	RF	XGBoost
1	MRW_TI	MRW_TI	MRW_T	cRNFL_G	cRNFL_G
2	MRW_G	MRW_G	MRW_TI	MRW_TI	mRNFL_I2
3	cRNFL_G	cRNFL_G	GCL_I1	mRNFL_I2	GCL_I1
4	GCL_I1	GCL_I1	mRNFL_I2	GCL_I1	MRW_TI
5	GCL_T1	GCL_T1	cRNFL_TS	cRNFL_TI	MRW_T
6	MRW_TS	MRW_TS	cRNFL_NS	GCL_I2	GCL_I2
7	mRNFL_I2	mRNFL_I2	cRNFL_G	MRW_G	MRW_G
8	MRW_NS	MRW_NS	GCL_T2	GCL_T1	cRNFL_TI
9	cRNFL_TI	cRNFL_TI	cRNFL_TI	GCL_T2	GCL_T1
10	MRW_NI	MRW_NI	rFT_33	MRW_NI	GCL_T2
11	cRNFL_TS	cRNFL_TS	MRW_G	rFT_28	MRW_TS
12	MRW_T	MRW_T	rFT_43	MRW_TS	MRW_NI
13	rFT_28	rFT_28	MRW_NS	MRW_T	cRNFL_TS
14	MRW_N	MRW_N	GCL_I2	rFT_38	IPL_T2
15	rFT_27	rFT_27	MRW_TS	rFT_17	rFT_28

CIT: conditional inference trees; LMT: logistic model tree; RF: random forest; XGBoost: extreme gradient boosting; AUC: area under the receiver operating characteristic (roc) curve
